# Symbiotic Bacteria System of *Locusta migratoria* Showed Antifungal Capabilities against *Beauveria bassiana*

**DOI:** 10.3390/ijms24043138

**Published:** 2023-02-05

**Authors:** Shuqian Tan, Hongshuang Wei, Ibrahima Camara, Haoran Jia, Kaili Cao, Wangpeng Shi

**Affiliations:** 1 Department of Entomology and MOA Key Laboratory of Pest Monitoring and Green Management, College of Plant Protection, China Agricultural University, Beijing 100193, China; 2 Institute of Medicinal Plant Development, Chinese Academy of Medical Sciences and Peking Union Medical College, Beijing 100193, China; 3 Department of Agriculture, Higher Agricultural and Veterinary Institute of Faranah Guinea, Faranah BP131, Guinea

**Keywords:** migratory locust, biological pest control, entomopathogenic fungi, symbiotic flora

## Abstract

The stability of symbiotic flora is an important indicator of the health of an organism. Symbiotic bacteria have been proven to be closely involved in the immune process of organisms. The pathogenicity of *Beauveria bassiana* was studied in relation to symbiotic bacteria on the surface and inside of the migratory locust (*Locusta migratoria*). The results showed that the surface disinfection of test locusts contributed to the pathogenicity of *B. bassiana* to locusts. Most of the surface bacteria of *L. migratoria* caused some inhibition of *B. bassiana* growth, and LM5-4 (*Raoultella ornithinolytica*), LM5-2 (*Enterobacter aerogenes*), and LM5-13 (*Citrobacter freundii*) showed the highest inhibitory effect on the growth of *B. bassiana.* The inoculation of locusts with additional surface symbiotic bacteria reduced the virulence of *B. bassiana* to *L. migratoria*. Infection by different strains of *B. bassiana* caused similar changes in the symbiotic flora of migratory locusts. The inoculation of locusts with additional intestinal symbiotic bacteria (*Enterobacter* sp.) reduced the virulence of *B. bassiana* to *L. migratoria*. These findings illustrate the effect of bacterial communities on fungal infections in *L. migratoria* when seen from the perspective of ecology in a microenvironment. The active antifungal substances of such bacteria and their mechanisms of action need further study.

## 1. Introduction

Since *Beauveria bassiana* was discovered as a pathogenic fungus of insects, the infection and toxicity mechanisms of *B. bassiana* in insects have been thoroughly studied [1,2]. *B. bassiana* kills its host in a succession of steps that include adhesion, germination, penetration, colonization, extrusion, and conidiogenesis [3]. *B. bassiana* has been established as a major biological pesticide for agricultural usage globally due to its broad host range and high infectivity [4,5,6]. 

However, the antifungal activity of the insect immune system as a defense against pathogenic fungi such as *B. bassiana* requires more exploration, and the variables influencing insect immunity to fungi must be studied from the standpoint of genetic evolution [7]. These immune systems involve both cellular immune responses and humoral immunity [8,9,10]. Insect immunity to pathogenic fungi severely limits the development of fungal biopesticides. 

Insects continuously interact with a diverse array of microorganisms in their environments, and certain bacteria can form life-long symbiotic partnerships with them [11]. The significance of the insect gut microbiota in the insect life cycle has been one of the most investigated of these interactions. Gut microbes contribute to insect fitness through nutrient digestion [12,13,14], hormone regulation [15], behavior patterns [16,17], and immunology [18]. However, research on the role of symbiotic bacteria in other regions of the body, particularly symbiotic bacteria on the integument, has been disregarded to some extent.

The interactions between insect symbiotic bacteria and invading microbes dictate the type of influence they have on the host [19]. Almost all interaction patterns known in macroscopic creatures, including rivalry and cooperation, may also be present in microbes [20]. Many studies have revealed that invasive microorganisms, particularly pathogens, must alter the host’s natural symbiotic microbiome’s homeostasis during colonization [21,22]. Thus, the host symbiotic flora may defend the host against invading microorganisms by preventing their colonization [22,23]. 

In addition to innate immunity, insects have developed ecological and behavioral immunity against pathogens [16,24], which is facilitated in part by a diverse set of microbe–host interactions. Many species employ microorganisms to protect themselves from predators, parasites, parasitoids, and diseases [22,25]. In order to demonstrate the role of symbiotic bacterial systems of *L. migratoria* in resisting invasive pathogenic fungi, we performed validation experiments in vitro and in vivo using some of the culturable bacteria of *L. migratoria*. In this paper, we provide a novel model of immunity in which insects employ their own symbiotic bacteria to fight invading fungi.

## 2. Results

### 2.1. Impact of Surface Disinfection of Test Insects

**Figure 1:**
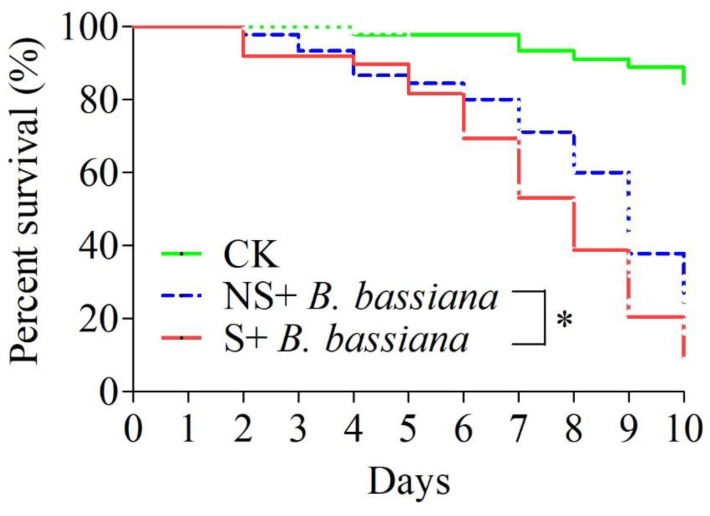
Effect of surface disinfection on survival of *Locusta migratoria* treated with *Beauvaria bassiana* strains (BbZJ1). CK = group of *Locusta migratoria* treated with 0.05% Tween-80. NS = no sterilization and S = sterilization. The log-rank (Mantel–Cox) test method in GraphPad 5 software was used to analyze the differences among the survival curves. “*” Significantly different at 0.05 level.

The survival rates of locusts inoculated with *B. bassiana* (strain BbZJ1) were compared between locusts that were locally sterilized (on the prothoracic sclerite, the site of inoculation) and unsterilized locusts as two treatments. After day 6 post-inoculation, survival rates became lower in the sterilized group (Figure 1). There was a significant difference in survival on day 10 between the two treatment groups (χ^2^ = 49.35, df = 2, *p* < 0.0001).

### 2.2. Bacteria Recovered from Surface of L. migratoria and Their Inhibition of B. bassiana Growth in Cultures

**Figure 2:**
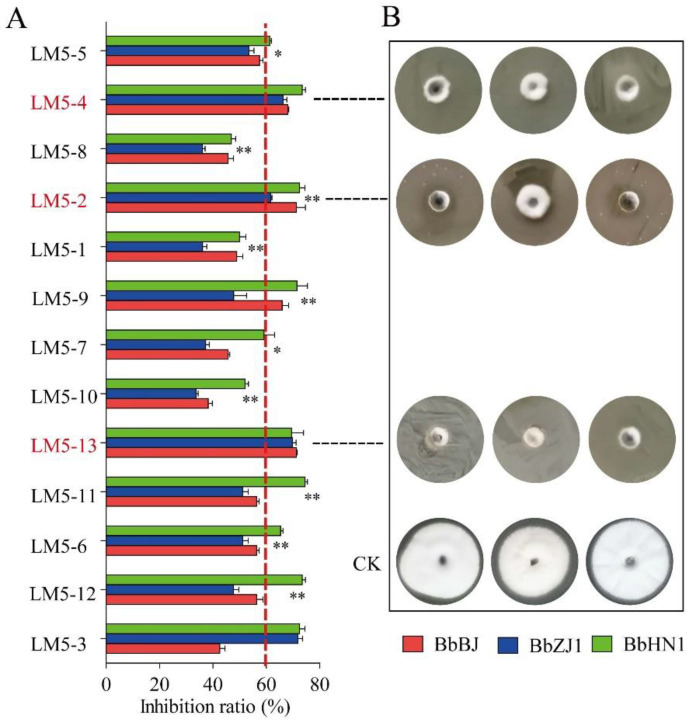
Inhibitory effect of migratory locust surface bacterium on *B. bassiana*. (**A**) Inhibition rates for *B. bassiana* strains by bacteria. Values (mean ± SE) with “*” or “**” are significantly different between strain BbZJ1 and other strains at a level of 0.05 or 0.01 (Kruskal–Wallis test). (**B**) *B. bassiana* colonies growing on medium coated with three of the recovered bacterial strains. CK: normal-growing and uninhibited fungal colonies.

Thirteen culturable bacterial strains were isolated from the body surface of migratory locusts from the test laboratory population and later identified. In our Petri dish assay, the inhibition of *B. bassiana* in plates with bacteria added reached 50% for most bacterial lines and more than 60% in a few of them (Figure 2A). Among the most inhibitory lines were the bacterial strains LM5-2, LM5-4, and LM5-13, which had inhibition rates of more than 60% against all three of the tested strains of *B. bassiana* (Figure 2A,B). The level of the growth inhibition of fungal strain BbZJ1 by most of the bacterial lines was lower than for the other two fungal strains (BbBJ and BbHN1) (Figure 2A,B). This was not the case, however, for three bacterial strains (LM5-3, 4, and 13). The colony diameters of *B. bassiana* BbZJ1 were significantly larger in the Petri dish fungal growth assay than those of the other two fungal strains after 10 days of growth on a medium coated with test bacteria, except for the treatments with bacterial strains LM5-4 and LM5-13 (Figure 2B). Notably, the LT_50_ of *B. bassiana* BBZJ1 against the migratory locust was the shortest among the three fungi, at 5.87 days (Table S1). The LT_50_ values of two other *B. bassiana* strains in migratory locusts were >7 days (Table S1). LM5-2, LM5-4, and LM5-13 were identified as *Enterobacter aerogenes*, *Raoultella ornithinolytica*, and *Citrobacter freundii*, respectively (Table S2).

### 2.3. Effect of Bacterial Supplementation on Fungal Virulence

**Figure 3:**
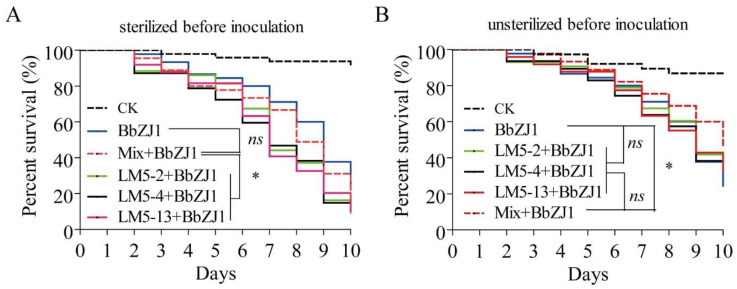
Effect of interaction between surface symbiotic bacteria and *Beauveria bassiana* on virulence of *B. bassiana* against *Locusta migratoria*. (**A**) Survival curves of *L. migratoria* that were surface-sterilized before inoculation with *B. bassiana* and bacteria. (**B**) Survival curves of locusts with unsterilized surfaces before inoculation with *B. bassiana* and bacteria. Mix: inoculation of a mixture of three bacteria strains. CK: treated with 0.05% Tween-80. The log-rank (Mantel–Cox) test method in GraphPad 5 software was used to analyze the differences among the survival curves. “*” Significantly different at 0.05 level. “*ns*” Not significant different at 0.05 level.

The pathogenicity of *B. bassiana* (strain BbZJ1) to locusts that had been surfaced-sterilized before bacterial supplementation was significantly increased compared to locusts with no bacterial supplementation following surface sterilization (Figure 3A). There was no significant difference between the mixed bacterial strain treatment (Mix + BbZJ1) and the control group (BbZJ1) (Figure 3A). However, in the absence of prior surface sterilization of the locusts, there were no significant differences among the single-strain bacterial treatments and the control group (BbZJ1). However, the mixed bacterial strain treatment group showed a significantly different increase in survival compared with the control group (Figure 3B).

### 2.4. Impact of Three Fungal Strains on Intestinal Bacteria of Locusts

Ten days after locust inoculation with each of our three *B. bassiana* strains, the total number of bacterial colonies recovered from sampling from the guts of locusts decreased significantly (Figure S1). The results of high-throughput RNA sequencing showed that infection by each of the three strains of *B. bassiana* caused changes in the taxonomic composition of the intestinal bacterial community in migratory locusts to various degrees.

**Figure 4:**
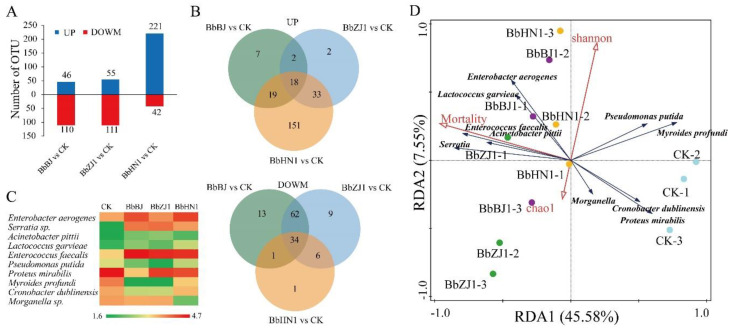
Changes in the intestinal bacteria in migratory locusts after *Beauveria bassiana* infection. (**A**) The number of Operational Taxonomic Units (OTUs) whose abundance increased or decreased in migratory locusts in response to infection by each of the three fungal pathogens. (**B**) Venn diagram showing how many OTUs increased or decreased in response to infection by all three fungal strains. (**C**) A heat map of the top ten most abundant bacteria. This figure plots the number of reads of 16 s DNA of related bacteria obtained by sequencing. The data used for analysis were calculated using Log10 (X). (**D**) Redundancy analysis of *L. migratoria* bacterial community.

Among the three fungi, infection by strain BbBJ caused the abundance of 46 bacteria to increase, while the abundance of 110 bacteria decreased. Strain BbZJ1 caused the abundance of 55 bacteria to increase, while the abundance of 111 bacterial OTUs (Operational Taxonomic Units) decreased (Figure 4A). Strain BbHN1 caused the most dramatic change, with 263 bacterial OTUs either rising (221) or falling (42) in abundance (Figure 4A). The abundance of 18 species increased for all three fungal strains, while 34 OTUs decreased (Figure 4B). Of the species of bacteria that changed (up or down) in abundance in response to fungal infection, the top five species that increased the most were *Enterobacter aerogenes, Serratia* sp., *Acinetobacter pittii*, *Lactococcus garvieae*, and *Enterococcus faecalis*, while the five that showed the largest decline in abundance were *Pseudomonas putida*, *Proteus mirabilis*, *Myroides profundi*, *Cronobacter dublinensis*, and *Morganella* sp. (Figure 4C). Redundancy analysis (Figure 4D) showed that locust mortality was not significantly correlated with either the Chao index or the Shannon index (Figure 4D). Moreover, the strengths of the two indices related to *B. bassiana* infection were not strong (Figure 4D). The position of each treatment point relative to mortality and bacterial variables (*Enterobacter aerogenes* in locusts suffering from sublethal fungal infection) indicated that the lower the levels of these bacteria, the higher the virulence of *B. bassiana* strains (Figure 4D).

### 2.5. Effect of Enterobacter sp. on Fungal Growth and Virulence

**Figure 5:**
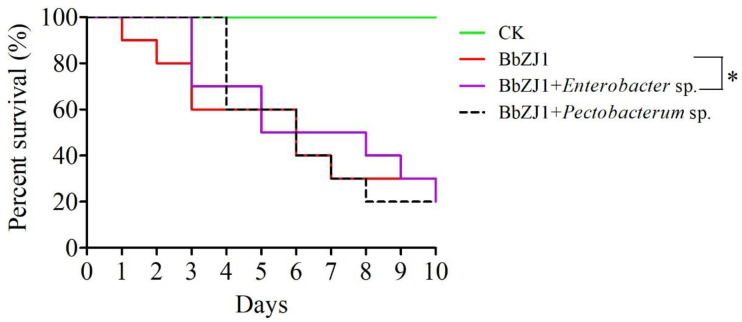
Effect of bacterial species on survival of *Locusta migratoria* co-inoculated with the bacterium and *Beauvaria bassiana* strain BbZJ1. The log-rank (Mantel–Cox) test method in GraphPad 5 software was used to analyze the differences among the survival curves. “*” Significantly different at 0.05 level.

The strain of bacteria (*Enterobacter* sp.) that showed the greatest increase in abundance during fungal infection was isolated and cultured (Figure S2A). We then used that bacterial strain as a supplement to the medium used to grow the test fungi to search for this bacterial strain’s inhibition of fungal growth. We found minimal growth of the fungi on the growth medium supplemented with *Enterobacter* sp., whereas fungal colonies grew normally on the medium supplemented with either *Escherichia coli* or *Pectobacterum* sp., two other bacteria we used as controls (Figure S2B). Conversely, *E. coli* did not grow on the fungus-laden medium, while there were significant areas of fungal growth inhibition near colonies of *Enterobacter* sp. (Figure S2C). The combined inoculation experiment also proved that the survival rate of migratory locusts inoculated with *B. bassiana* and bacteria (*Enterobacter* sp.) was significantly higher than when locusts were inoculated only with *B. bassiana* (Figure 5 and Figure S3)*. Pectobacterum* sp. showed no inhibition of *B. bassiana* virulence (Figure 5 and Figure S3).

## 3. Discussion

A stable microbial community is critical for organism health [26]. The microbial communities on living organisms are always challenged by both invasive [21] and opportunistic pathogens [27]. *Beauveria bassiana* is an invasive species, in contrast to the normal migratory locust microbial community in the microenvironment of the locust. By analyzing the symbiotic bacterial community on the surface and inside the body of migratory locusts, we found that the normal bacterial community in locusts affected the infection and pathogenicity of *B. bassiana* and that several of these key bacteria with antifungal activity can protect migratory locusts to some extent from *B. bassiana*.

The virulence of different strains of the same pathogenic fungi to the same host can sometimes be very different [28]; just like the results obtained in the LT50 test, the virulence of strain BbZJ1 to locusts was significantly stronger than that of strains BbBJ and BbHN1. Changes in virulence factors in fungi due to gene mutation [29,30] and immune differences among host individuals possibly explain this variation in virulence [27,31]. Previous studies have shown that microbial communities play key roles in the health and development of their multicellular hosts and are an important part of the arthropod immune system [27,32].

The antagonistic relationship between bacteria and fungi makes it possible for insects to use symbiotic bacteria to resist infection by pathogenic fungi. *Burkholderia* symbionts, for example, provide nutritional benefits and resistance against insecticides in stinkbugs, defend *Lagria* beetle eggs against pathogenic fungi, and may be involved in nitrogen metabolism in ants [11]. We believe that the resistance to fungi provided by entomic symbiotic bacteria is the result of community homeostasis. 

By comparing previous studies, we found that locusts do not have a relatively stable structure of the community of intestinal flora, which is variable [21,22,33] because of their variable living conditions and food. It was reported that intestinal bacteria with higher abundance at the genus level were *Weissella*, *Lactococcus*, *Citrobacter*, *Raoultella*, and *Enterococcus* [21,22]. In addition, the variation in the abundance of *Weissella* sp. was closely related to infection by *B. bassiana*, which implied resistance to infection by *B. bassiana* [22]. To our confusion, *Weissella* was not detected in our results, but *Enterococcus*, *Proteus*, *Providencia*, *Klebsiella*, *Myroides*, and so on were identified. Fortunately, our study also proved that the *Enterobacter* sp. strain isolated from locusts had a certain inhibition ability against *B. bassiana*.

Evidence exists for competition between some fungi and bacteria because of the overlap of their ecological niches [34]. Certain bacteria can inhibit fungi, such as *Enterobacter aerogenes* and *Bacillus subtilis*, which produce substances with inhibitory effects on *Phytophthora cactorum* [35,36]. In this study, we isolated a strain of *Enterobacter aerogenes* from the migratory locust surface that could inhibit the growth of the entomopathogenic fungi *B. bassiana*. Moreover, the isolate of *Enterobacter* sp. inside of the locust can also significantly reduce the virulence of *B. bassiana* to locusts. However, this paper only introduces the effect of the locust’s bacterial community on its antagonistic fungi from the perspective of ecology. The active antifungal substances and their mechanism need further study.

In the process of studying the protection of symbiotic bacteria toward *Monochamus alternatus*, it was found that *Pseudomonas* and *Serratia* bacteria showed strong inhibitory activity against *B. bassiana* by reducing fungal conidial germination and growth rather than regulating host immunity [37]. Here, our experiments demonstrated that the bacteria *Enterabacter aerogenes*, *Raoultella ornithinolytica,* and *Citrobacter freundii* on the body surface were responsible for the reduced virulence of *B. bassiana* against the locust. Additionally, *Enterabacter* sp. inside the body significantly inhibited the growth of *B. bassiana*. 

In conclusion, we took *Locusta migratoria* and *Beauveria bassiana* as study objects and observed that the symbiotic bacteria of *L. migratoria*, such as *E. aerogenes*, *Raoultella ornithinolytica*, and *Citrobacter freundii*, inhibited the growth of *B. bassiana* in in vitro experiments. The mortality of locusts infected with *B. bassiana* was dramatically reduced following extra supplementation of these cultivable symbiotic bacteria. These results demonstrated that native bacterial systems in the microenvironment of the insect surface and interior defend against an invasive pathogenic fungus. In particular, the *Enterobacter* sp. both on the surface and within the bodies of *L. migratoria* inhibited *B. bassiana*. Indeed, research on the antagonism of insect symbiotic bacterial systems against harmful fungi is important in two ways. First, the antifungal bacteria on insects considerably diminish the action of biocontrol fungi on pests; thus, we need to make efforts to mitigate the effect of antagonism while producing fungal insecticides. However, many commercial insects, such as silkworms and bees, are frequently plagued by pathogenic fungi and suffer from large-scale population mycosis, significantly reducing their contribution to humans. As a result, antifungal microorganisms can preserve these economically important insects. Our findings have crucial implications for how to exploit the antagonistic relationship between symbiotic bacteria and pathogenic fungi to manage pests and safeguard beneficial insects.

## 4. Materials and Methods

### 4.1. Insects and Microorganisms

The locusts, *Locusta migratoria*, used here were obtained from a laboratory colony maintained by the Key Bio-control Laboratory for Locusts at the China Agricultural University, Beijing. Locusts had been reared in a stable external environment for more than ten generations. Nymphs were reared in cages (50 cm height and 15 cm diameter) in the laboratory under a long-day photoperiod (16 h light/8 h darkness cycle) at 30 ± 2 °C and 75% relative humidity. Insects were fed with wheat leaves, and cages were cleaned daily [21].

In this experiment, several bacterial strains were isolated from the body surface and internal tissues of the migratory locust. Three strains of *B. bassiana* (BbBJ, BbZJ1, and BbHN1; host pest: *L. migratoria*) that were stored in the laboratory of Biological Control of Agriculture Pest, China Agricultural University [38], were used for experiments. *B. bassiana* strains were activated with PDA medium at 28 °C. The bacterial strains were cultured on LB solid medium. 

### 4.2. Calculation of LT_50_ Values for the Three Fungal Strains

LT_50_ values (time to 50% mortality) were calculated for each of the three *B. bassiana* strains (BbBJ, BbZJ1, and BbHN1). To calculate LT_50_ values, the third-instar nymphs of *L. migratoria* were submerged in a fungal suspension with 0.05% Tween 80 (10^8^ viable conidia/mL) for 30 s. We used a completely randomized design with three replicates (*n* = 20 for each replicate). After inoculation via a water bath of spores, locusts were held in an incubator (as described above) and fed wheat leaves. Mortality was recorded daily and used to compute LT_50_ values. 

### 4.3. Collection and Identification of Symbiotic Bacteria

Sterile cotton swabs were used to remove food debris and other foreign matter from the body surfaces of test locusts (5th instar). Thirty cleaned locusts were then placed in sterile water and scrubbed gently one by one. The bathwater was collected and spread on LB solid medium and then cultured at 37 °C for 24 h. Single colonies were selected and transferred into an LB liquid medium and incubated at 37 °C for 12 h.

The intestines of 5th-instar locusts were taken and crushed in 2 mL of sterile water for the recovery of intestinal bacteria from locusts. The debris was filtered out using sterile gauze. The remaining suspension was collected and spread on an LB solid medium and cultured at 37 °C for 24 h. Single colonies were selected and transferred into an LB liquid medium and incubated at 37 °C for 12 h.

### 4.4. DNA Sequencing of Recovered Bacteria

To obtain DNA sequences from the bacteria recovered from locusts, the V3–V4 hypervariable region of the bacterial 16S ribosomal RNA (rRNA) gene was amplified with the primers 338F (5′-ACTCCTACGGGAGGCAGCAG-3′) and 806R (5′-GGACTACNNGGGTATCTAAT-3′). PCR was then carried out on a Mastercycler Gradient (Eppendorf, Hamburg, Germany) using 50 μL reaction volumes containing 5 μL of 10 × Ex Taq Buffer (Mg2+ plus), 4 μL of 12.5 mmol/L deoxynucleotide triphosphate mix (each), 1.25 U Ex Taq DNA polymerase, 2 μL of template DNA, and 36.75 μL of double-distilled H_2_O. The cycling parameters were 94 °C for 2 min, followed by 30 cycles of 94 °C for 30 s, 57 °C for 30 s, and 72 °C for 30 s, with a final extension at 72 °C for 10 min. Three PCR products per sample were collected and then pooled to mitigate reaction-level PCR biases. The PCR products were purified using a QIAquick Gel Extraction Kit (QIAGEN, Dusseldorf, Germany) and sequenced by the Sangon Biotech Company, Shanghai, China.

### 4.5. Virulence Testing of Natural vs. Sterilized Locust Body Surfaces

To obtain sterilized locust body surfaces, the prothoracic sclerite of the locust was wiped with 75% alcohol. Then, the sterilized surfaces were inoculated with 10 μL of a *B. bassiana* spore suspension (1.6 × 10^8^ spores/mL). Unsterilized locusts (5th instar) were infected with *B. bassiana* (BbZJ1) as a positive control. Sterile water was applied to the prothoracic sclerite as the general control. Each treatment was repeated 3 times with 20 5th-instar nymphs as one replicate. Locusts were reared under the conditions mentioned above. The number of dead locusts was recorded daily.

### 4.6. Microbial Growth Inhibition Test

The 13 symbiotic bacteria recovered from the body surfaces of locusts were incubated with an LB liquid medium at 37 °C for 24 h. The suspension of cultured bacteria was spread evenly on a beef extract peptone solid medium. A hole 5 mm in diameter was cut into the middle of the plate of the medium. Ten microliters of each of the three *B. bassiana* strains (BbBJ, BbZJ1, and BbHN1) at a 10^4^ spores/mL concentration was placed in the hole. The plates of the treated solid medium were incubated at 37 °C for 7 days, and the diameters of the fungal colonies were measured and recorded 10 days after inoculation.

For the bacteria recovered from the intestines of locusts, microbial growth inhibition tests were performed in the following manner. Fifty-microliter suspensions of the *Pectobacterum* sp. and *Enterobacter* sp. were each placed on a beef extract peptone solid medium coated with the fungus *B. bassiana* BbZJ1; a 50 μL suspension of *B. bassiana* BbZJ1 was placed on a beef extract peptone solid medium coated with the *Pectobacterum* sp. or *Enterobacter* sp. Then, the growth of the fungi and bacteria was observed. The concentration of both the fungus and the bacteria was 10^4^ spores/mL. *Escherichia coli* was used as a negative control.

### 4.7. Effect of Bacterial Supplementation on Fungal Virulence

The virulence of one strain of *B. bassiana* (BbZJ1) to locusts treated with bacterial supplementation with three bacterial strains, separately or together as a mixture, was assessed. Before fungal and bacterial inoculation, the prothoracic sclerites of locusts were either pre-sterilized or not. For tests with bacteria collected from the outside of locust bodies, we evenly applied 10 μL of a suspension of three recovered bacterial lines (LM 5-2, LM 5-4, and LM 5-13) or each strain as a separate treatment at 10^5^ bacterial spores/mL. Then, fungal spores were applied. Locusts receiving treatments had either natural bacterial flora or were locusts whose prothoracic sclerite had been sterilized.

For tests with bacteria recovered from intestinal tissue, locusts were fed wheat leaves treated with *Pectobacterum* sp. or *Enterobacter* sp. (10^5^ spores/mL) after the locusts were first inoculated with *B. bassiana* on the prothoracic sclerite. Locusts in this group were fed bacteria on wheat leaves daily. Each treatment was repeated 3 times with 20 fifth-instar nymphs as one replicate. Locusts were then reared under the same conditions mentioned above, and the number of dead locusts was recorded daily.

### 4.8. Calculation of LT_50_ Values for the Three Fungal Strains

LT_50_ values (time to 50% mortality) were calculated for each of the three *B. bassiana* strains (BbBJ, BbZJ1, and BbHN1). To calculate LT_50_ values, third-instar nymphs of *L. migratoria* were submerged in a fungal suspension (10^8^ viable conidia/mL) for 30 s. We used a completely randomized design with three replicates (*n* = 20 for each replicate). After inoculation via a water bath of spores, locusts were held in an incubator (as described above) and fed wheat leaves. Mortality was recorded daily and used to compute LT_50_ values. 

### 4.9. Impact of Three Fungal Strains on Intestinal Bacteria of Locusts

High-throughput sequencing was used to examine the impact of infection by three species of *B. bassiana* (BbBJ, BbZJ1, and BbHN1) on the intestinal bacteria of locusts. Third-instar locusts were inoculated with a low dose (1 × 10^4^) with one of the three tested strains of *B. bassiana* separately and incubated for 10 days. The control was locusts not inoculated with fungi. Ten intestines of locusts infected with fungi were mixed to extract DNA in each treatment. The V4–V5 hypervariable region of the bacterial 16S rRNA gene was amplified. The PCR products of the V4–V5 region of the bacterial 16S rRNA gene were sequenced using the Roche 454 FLX Titanium sequencer (Roche, Nutley, NJ, USA) at Allwegene Company (Beijing, China). The samples were individually barcoded to enable multiplex sequencing [21]. The experiment was repeated in three replicates. 

After the run, image analysis, base calling, and error estimation were performed using Illumina Analysis Pipeline Version 2.6. The optimized sequences were clustered into Operational Taxonomic Units (OTUs) with a similarity cut-off of 97% using Uparse (version 7.0.1090). Ribosomal Database Project (RDP) Classifier software [39] was used to classify the sequences, and barcodes were searched in the NCBI database. The effective sequences were then assigned to NCBI taxonomies with molecular evolutionary genetics analysis (MEGAN) [40]. 

### 4.10. Statistical Analyses

Based on species annotation and relative abundance results, R (v3.6.0) software was used to analyze the histogram of species composition and perform redundancy and partial least-squares discrimination analyses. A linear discriminant effect size analysis was performed using Phython (v2.7) software. The LT50 values of *B. bassiana* against *L. migratoria* were analyzed using probit analysis in SPSS 16 software. The log-rank (Mantel–Cox) test method in GraphPad 5 software was used to analyze the differences among the survival curves. The Kruskal–Wallis test was conducted to see the differences in the inhibition rates for *B. bassiana* strains by bacteria.

## Data Availability

The amplicon data in this study have been deposited into the CNGB Sequence Archive (CNSA) of the China National GeneBank DataBase (CNGBdb) with accession number CNP0003870.

## Supplementary Materials

The following supporting information can be downloaded at https://www.mdpi.com/article/10.3390/ijms24043138/s1.
