# Supplementary material for: Symbiotic Bacteria System of Locusta migratoria Showed Antifungal Capabilities against Beauveria bassiana
Source: Int J Mol Sci. 2023 Feb 5;24(4):3138. doi: 10.3390/ijms24043138 (PMC9965112; doi:10.3390/ijms24043138)
Supplement: Supplementary file 1 [file ijms-24-03138-s001.zip › ijms-2177556-supplementary.pdf]

Supplementary information

Symbiotic bacteria system of *Locusta migratoria* showed antifungal capabilities against *Beauveria bassiana*

Shuqian Tan[1]*, Hongshuang Wei[2], Ibrahima Camara[1, 3], Haoran Jia[1], Kaili Cao[1], Wangpeng Shi[1]*,

1. Department of Entomology and MOA Key Laboratory of Pest Monitoring and Green Management, College of Plant Protection, China Agricultural University, Beijing, China

2. Institute of Medicinal Plant Development, Chinese Academy of Medical Sciences and Peking Union Medical College, Beijing, China

3. Department of Agriculture, Higher Agricultural and Veterinary Institute of Faranah Guinea, Faranah, Guinea

*Corresponding authors: Shuqian Tan, E-mail: zhiweijianniyixiao@163.com; Wangpeng Shi, E-mail: wpshi@cau.edu.cn

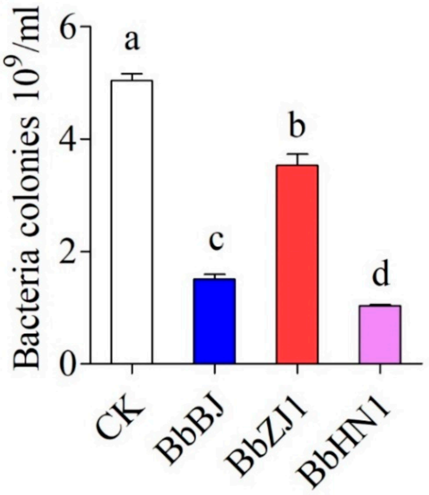

Figure S1. **Effects of *Beauveria bassiana* on bacterial abundance in *L. migratoria*.**

Means ± SE within a column followed by the same upper letter were not statistically different ($p < 0.05$).

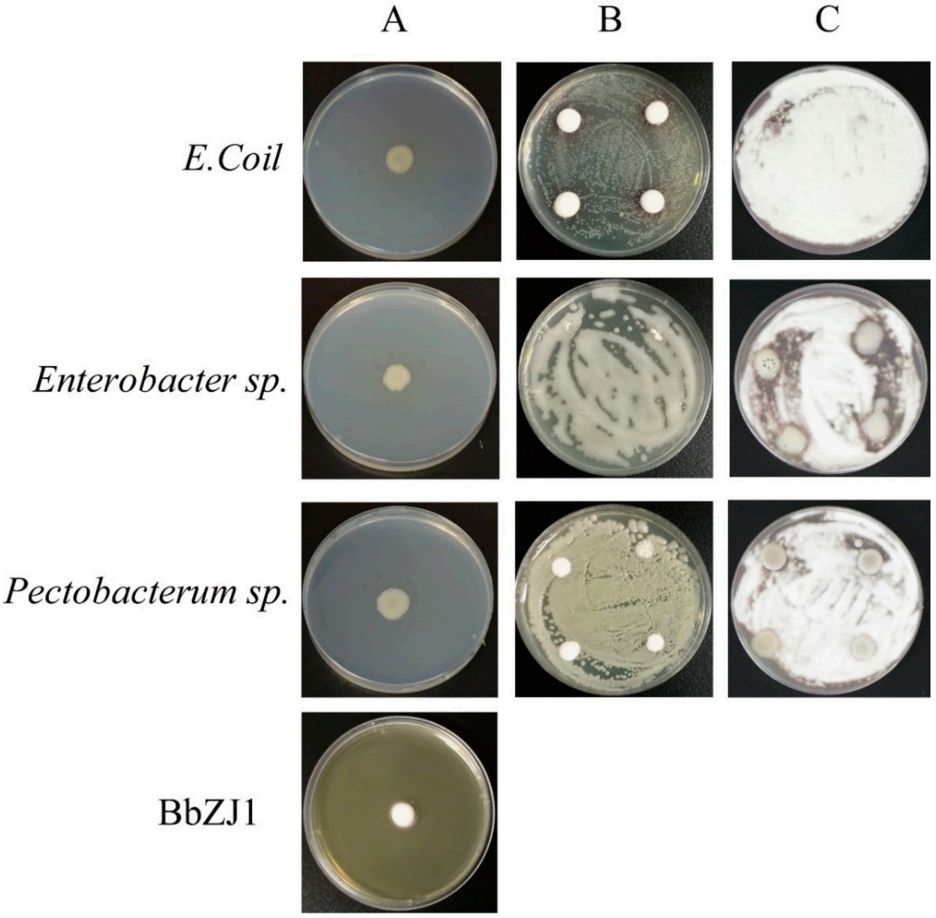

Figure S2 Inhibition of growth of *Beauveria bassiana* by bacteria.

A. Independent colonies of bacteria or *B. bassiana*. B. Growth of *B. bassiana* on a culture medium filled with bacteria. C. Growth of bacteria on a culture medium filled with *B. bassiana*.

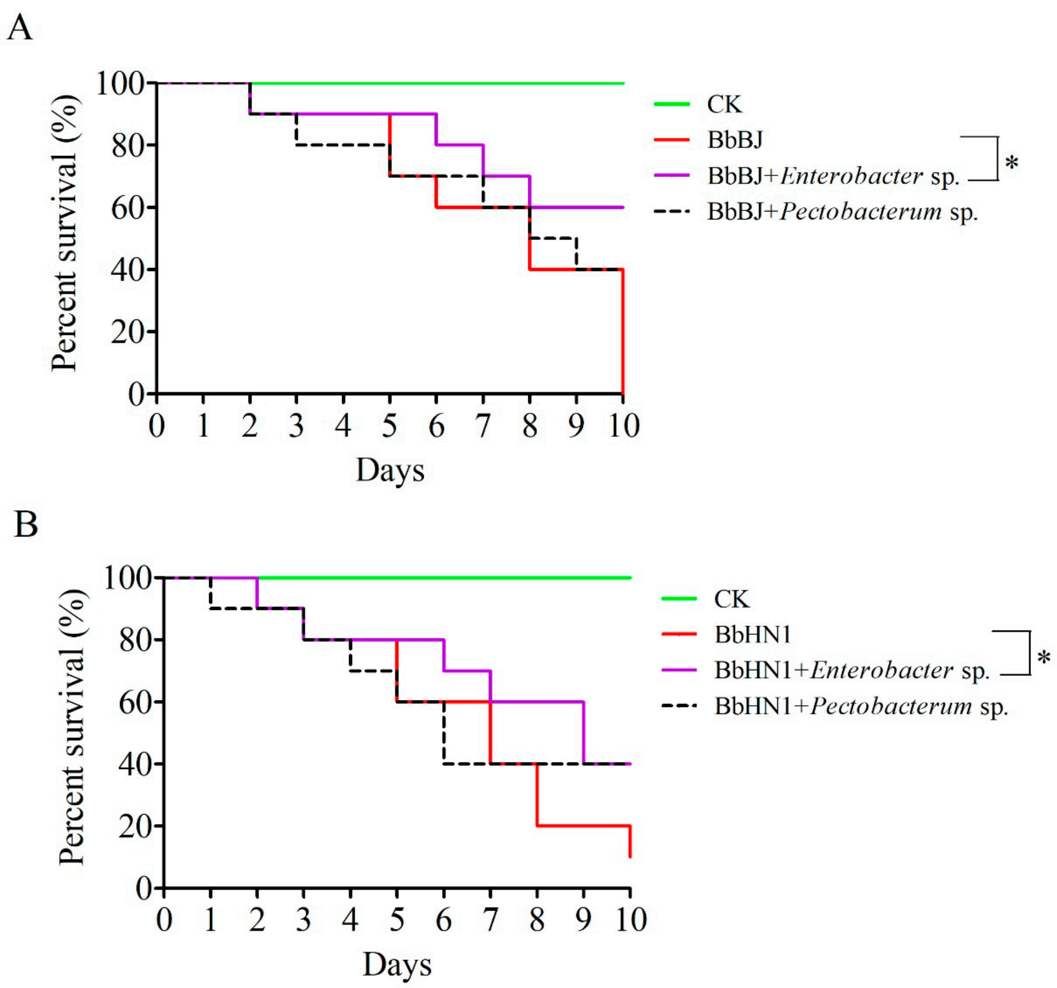

Figure S3 Effect of vivo bacteria of *Locusta migratoria* and *Beauvaria bassiana* strains (BbBJ and BbHN1) co-inoculation on locust survival.

The log-rank (Mantel-Cox) test method in GraphPad 5 software was used to analyze the differences among the survival curves. "*" is significantly different at 0.05 level.

Table S1. LT_{50} of the three regionally *B. bassiana* strains against locust nymphs.

Strains	LT_{50} (d)
BbBJ	7.17±0.15b
BbZJ1	5.87±0.34a
BbHN1	7.86±0.39b

Note: Means ± SE within a column followed by the same upper letter were not statistically different ($p < 0.05$). The LT50 were analyzed using the probit analysis in SPSS 16 software.

Table S2. Reference sequence information.

Strains	Species	GenBank	Percent identity (%)
LM 5-2	*Enterobacter aerogenes*	LC066142.1	99.54
LM 5-4	*Raoultella ornithinolytica*	MT568560.1	99.31
LM 5-13	*Citrobacter freundii*	MK299996.1	99.31
